# Live Cell Imaging of Germination and Outgrowth of Individual *Bacillus subtilis* Spores; the Effect of Heat Stress Quantitatively Analyzed with SporeTracker

**DOI:** 10.1371/journal.pone.0058972

**Published:** 2013-03-25

**Authors:** Rachna Pandey, Alex Ter Beek, Norbert O. E. Vischer, Jan P. P. M. Smelt, Stanley Brul, Erik M. M. Manders

**Affiliations:** 1 Molecular Biology and Microbial Food Safety, Swammerdam Institute for Life Sciences, University of Amsterdam, Amsterdam, The Netherlands; 2 Van Leeuwenhoek Centre for Advanced Microscopy Section of Molecular Cytology, Swammerdam Institute for Life Sciences, University of Amsterdam, Amsterdam, The Netherlands; 3 Department of Molecular Biotechnology, Faculty of Bioscience Engineering, University of Ghent, Ghent, Belgium; Loyola University Medical Center, United States of America

## Abstract

Spore-forming bacteria are a special problem for the food industry as some of them are able to survive preservation processes. *Bacillus* spp. spores can remain in a dormant, stress resistant state for a long period of time. Vegetative cells are formed by germination of spores followed by a more extended outgrowth phase. Spore germination and outgrowth progression are often very heterogeneous and therefore, predictions of microbial stability of food products are exceedingly difficult. Mechanistic details of the cause of this heterogeneity are necessary. In order to examine spore heterogeneity we made a novel closed air-containing chamber for live imaging. This chamber was used to analyze *Bacillus subtilis* spore germination, outgrowth, as well as subsequent vegetative growth. Typically, we examined around 90 starting spores/cells for ≥4 hours per experiment. Image analysis with the purposely built program “SporeTracker” allows for automated data processing from germination to outgrowth and vegetative doubling. In order to check the efficiency of the chamber, growth and division of *B. subtilis* vegetative cells were monitored. The observed generation times of vegetative cells were comparable to those obtained in well-aerated shake flask cultures. The influence of a heat stress of 85°C for 10 min on germination, outgrowth, and subsequent vegetative growth was investigated in detail. Compared to control samples fewer spores germinated (41.1% less) and fewer grew out (48.4% less) after the treatment. The heat treatment had a significant influence on the average time to the start of germination (increased) and the distribution and average of the duration of germination itself (increased). However, the distribution and the mean outgrowth time and the generation time of vegetative cells, emerging from untreated and thermally injured spores, were similar.

## Introduction

Spore-forming bacteria are an aggravating problem for the food industry and public health. For example, spores of Gram-positive bacteria such as *Bacillus* and *Clostridium* cause food spoilage and food borne diseases [1], [2], [3]. The metabolically dormant spores are extremely resistant to environmental stresses [4], [5]. Some may even survive harsh preservation treatments that are commonly used in some of the industrial processes. In contrast, the metabolically active form of the bacterium, the vegetative cell, is much easier to kill than the dormant spore. Therefore, it would be advantageous to deliberately drive spores in food into their vegetative form in order to facilitate their inactivation with relatively mild food preservation treatments. In practice this should be done by triggering spores with germinants or physical treatments that allow their ‘rapid return to life’ through the process of germination and outgrowth [6]. However, the timing of this process is difficult to predict accurately as spores are generally seen to germinate at different times and at different rates. Consequently, this heterogeneity makes predictions of microbial stability of food products exceedingly challenging [2], [7].

To get to a better understanding of heterogeneous germination and outgrowth, time-resolved single spore studies are essential. There have been few reports on spore germination at the single spore level. Using a combination of Raman tweezers and differential interference contrast microscopy, the labs of Peter Setlow and Yong-qing Li have investigated germination and coinciding release of Ca^2+^-dipicholinic acid (CaDPA) of individual spores of different *Bacillus* species [8], [9], [10]. They observed that both the time to the start of CaDPA release (start of germination) and the time period of CaDPA release (germination speed) increased in wet-heat-treated spores. Although the germination process was analyzed in great detail, subsequent outgrowth and vegetative growth were not investigated.

A study in which both non-treated as well as thermally-treated wild-type *Bacillus subtilis* spores were sorted individually in 96-wells of micro titer plates after which individual wells were monitored over time for vegetative growth under product-relevant conditions showed significant heterogeneity [11]. Whilst the timing of germination, the germination duration itself, the time to first vegetative doubling, as well as the initial generation times could not be analyzed separately in this study, the method gave clear insight in the resulting heterogeneity of the sum of the individual phases. In order to de-convolute the process in these individual elements and thus enhance our mechanistic insight in the regulation of spore ‘awakening’, live imaging techniques are required. Here we describe the development of such a system.

Phase-contrast microscopy allows us to observe at the single spore level the process of germination by assessing the transition of spores from phase-bright to phase-dark, their outgrowth by measuring the time interval between the phase-dark formation and the first cell division, as well as vegetative cell divisions for every individual cell of a population. Stringer *et al*. [12], [13], [14] used phase-contrast microscopy to determine the relation between different stages in germination and subsequent outgrowth of spores from the anaerobically growing nonproteolytic *Clostridium botulinum* strain. Their conclusion was that the distribution of the times to germination as well as outgrowth and subsequent growth, showed considerable increased variability. All stages contribute to the overall variability in the observed lag-time with the time to germination being most affected by a thermal stress.

In a recent study, de Jong *et al.* [15] presented a method to mount cells from the aerobic spore former *Bacillus subtilis* on a microscope slide and study their sporulation. The authors reported only qualitative details on the growth of vegetative cells. Although, in principle their method could be used to study the dynamics of growth and division of vegetative cells as well as germination and outgrowth of spores, their paper did not address these interesting aspects. In addition, no quantitative validation of vegetative cell growth under the condition for live imaging versus those prevailing in well-aerated shake flasks was performed.

In this paper, we propose a novel phase-contrast microscopy chamber that allows us to study at the individual cell level the real time dynamics of spore germination, outgrowth, and vegetative growth of aerobically growing *B. subtilis*. This closed air-containing chamber can optionally be combined with fluorescence microscopy. The efficiency of our chamber was assessed by measuring generation times of vegetative *B. subtilis* cells growing in the chamber and comparing the values to those obtained for cultures growing in conventional well-aerated shake flasks. Spore germination and outgrowth after applying a wet-heat stress of 10 min 85°C was also assessed in the proposed chamber. Additionally, we present an image analysis tool, called “SporeTracker”, for a detailed (semi-automated) analysis of spore germination, outgrowth, and vegetative growth. This work builds on preliminary experiments [16] that we now elaborated in full describing all technologies including the development of SporeTracker and the analysis of a specific thermal stress condition. The heat treatment significantly delayed the average start of germination and significantly increased the average time of germination itself. However, the outgrowth time and the generation time of vegetative cells, emerging from untreated and thermally injured spores, did not seem to be significantly affected. We demonstrate that our closed air-containing chamber and image analysis tool is suitable for the quantification of heterogeneous spore germination and outgrowth of aerobic *B. subtilis*.

## Materials and Methods

### Strain and Growth, Sporulation, and Germination Conditions

The strain used in this study was *B. subtilis* 168 laboratory wild-type strain 1A700 (*trpC2*).

Exponentially growing cells were prepared from a single colony in shake flasks in either undefined rich media: tryptic soy broth (TSB) and Luria-Bertani (LB), or in a defined minimal medium as described previously [17]. The defined minimal medium was buffered with 3-(*N*-morpholino)propanesulfonic acid (MOPS) to pH 7.4 (hereafter referred to as MOPS medium). As carbon and nitrogen-sources, 10 mM glucose and 10 mM NH_4_Cl were used. The optical density at 600 nm (OD_600_) was assessed in time to check whether the cells were in the exponential phase. Cells in the early exponential growth phase (OD_600_ = 0.2) were used for shake flask and time-lapse microscopy experiments (see below). The growth rate of liquid cultures grown in shake flasks was calculated by measuring the OD_600_ in time from three biological independent experiments.

Spores were prepared in defined MOPS medium and harvested as described recently [18]. Sporulation was induced by glucose exhaustion and allowed for 96 h during which its efficiency was followed using phase-contrast microscopy. The harvested spore crop contained >99.9% of phase-bright spores and were stored in distilled water at 4°C. All spores used for germination and outgrowth experiments were first heat-activated in distilled water for 30 min at 70°C. Germination and outgrowth of heat-activated spores was triggered in MOPS medium supplemented with 10 mM L-asparagine, 10 mM glucose, 1 mM fructose, and 1 mM potassium chloride (AGFK). Wet-heat inactivation of spores was assessed using the screw-cap tube method used by Kort *et al.* [17]. In short, a 1 ml (heat-activated) spore suspension was injected with a Hamilton syringe into a preheated metal screw-cap tube containing 9.0 ml of distilled water. The tube was heated by immersing it completely in a glycerol bath (85°C for 10 min). After 10 min the tube was immediately transferred to ice/water. Heat-inactivated and untreated spore suspensions were used for time-lapse microscopy as described below.

### Slide Preparation

**Figure 1:**
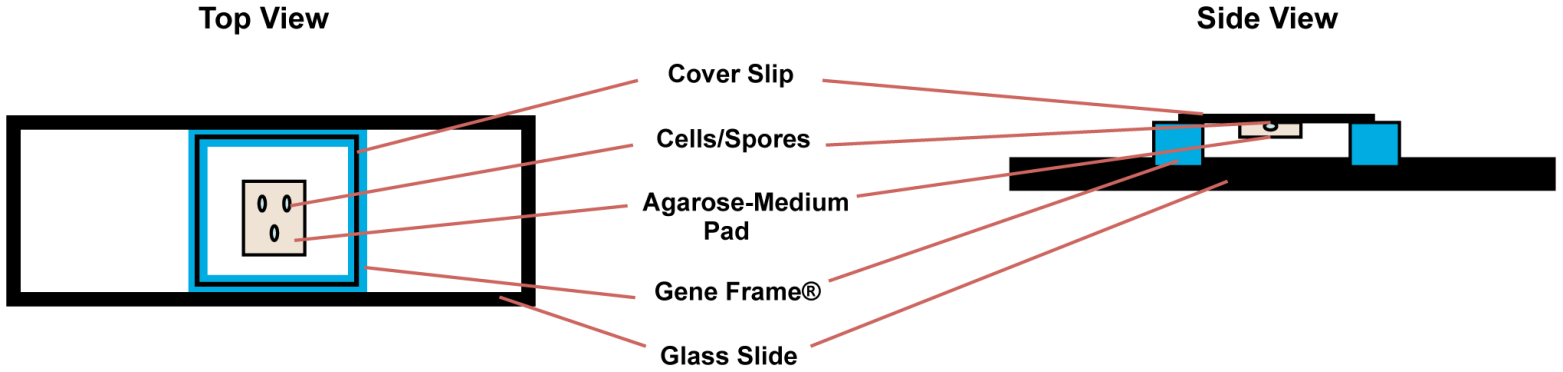
Schematic picture of the top and side view of the designed closed air-containing chamber for live cell imaging. A chamber was prepared by attaching a Gene Frame® to a standard microscope slide and cover slip. A thin, semisolid matrix pad (160 µm) of 1% agarose – medium was made. The pad was loaded with exponentially growing vegetative cells or heat-activated spores. The cover slip was placed in upside down position onto the Gene Frame® (See Materials and Methods for details).

A closed air-containing chamber, shown in Figure 1, was prepared by attaching a 65 µl volume Gene Frame® (1.5×1.6 cm, Thermo Scientific) to a standard microscope slide. A thin (∼160 µm), semisolid matrix pad (1×1 cm) with different concentrations of growth medium was made by adding 1% agarose (Sigma - Aldrich) to the medium in a 1∶1 ratio and spreading the final solution onto a siliconized glass cover-slip (24×32 mm, Thermo Scientific). Exponentially growing vegetative cells or spores (1 µl) were loaded onto the pad and then the pad was transferred upside down onto another glass cover slip (18×18 mm, Thermo Scientific). This glass cover slip was placed on the Gene Frame® and pressure was applied on the cover slip along the Gene Frame® for complete sealing. The resulting chamber was used for time-lapse microscopy.

### Time-lapse Microscopy

Time-lapse series were made by making use of a temperature-controlled boxed incubation system for live imaging set at 37°C and observing the specimens with a 100X/1.3 plane Apochromatic objective (Axiovert-200 Zeiss, Jena, Germany). Images were taken by a CoolSnap HQ CCD camera (Roper Scientific), using Metamorph software 6.1 (Molecular Devices). Phase-contrast time-lapse series were recorded at a sample frequency of 1 frame per 30 s or 1 min for 4 to 5.5 h, depending on the experiment. Maximally 9 different fields of view were recorded in parallel per experiment. In each field of view, on average 12 isolated vegetative cells or spores were identified and followed in time. This resulted in approximately 90 cells/spores at the start of imaging per experiment being analyzed. Three biological replicates and maximally nine technical replicates (recorded fields of view on one slide) were performed for each condition.

### Analysis with SporeTracker

**Figure 2:**
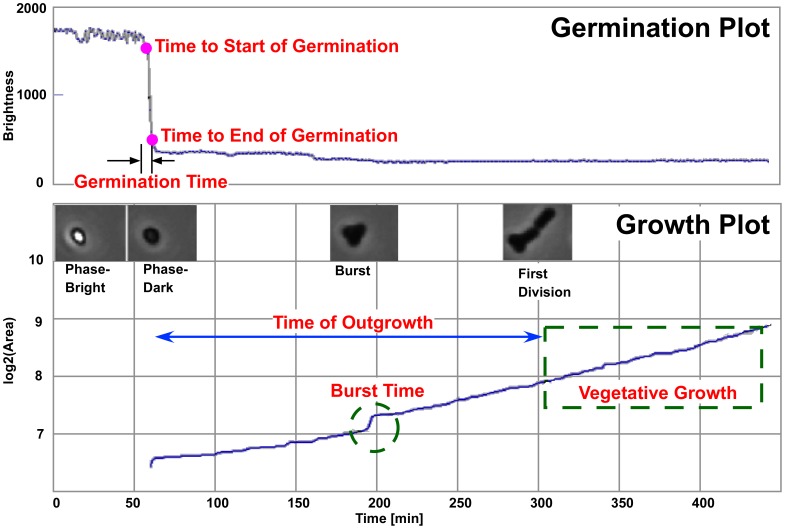
Dual plot showing spore germination and outgrowth of one heat-activated *B. subtilis* 1A700 spore as analyzed with SporeTracker. Above: Phase-bright to phase-dark transition, marked with a small circle at 90% (start of germination) and 10% (end of germination) of the entire (pixel) intensity drop range (brightness). Below: various snapshots at different stages of germination and outgrowth. The exponential growth phase (appearing linear in the log_2_ transformed plot of the measured area) is used to calculate the generation time. The burst of the cell out of the spore coat is accompanied by a relative short and significant increase in area (marked by the green circle). In SporeTracker, the cursor can be dragged across such a plot to observe live the various phases of spore germination and outgrowth and subsequent growth of the corresponding cell.

The phase-contrast microscope recorded the complete sequence of spore germination, outgrowth and cell divisions of bacteria emerging from spores. *B. subtilis* spores appeared as bright spots. As they germinated, their microscopic appearance became phase-dark. To follow the germination and outgrowth process, and subsequent cell division in time, the decrease in pixel intensity and increase in area were analyzed, respectively. To that end, “SporeTracker” <http://simon.bio.uva.nl/objectj/examples/sporetracker/SporeTracker.htm> was developed. This macro runs in combination with ObjectJ <http://simon.bio.uva.nl/objectj>, which is a plugin for ImageJ <http://rsb.info.nih.gov/ij>. SporeTracker is configured to measure the time to start of germination, the germination time itself ( = duration of phase-bright to phase-dark transition), the outgrowth time (from phase-dark to first division), as well as growth rates of vegetative bacteria emerging from the spores in any desired time frame; the program generates the corresponding plots (Figure 2) and numerical output from any number of movies. An important feature of SporeTracker is that the growth plots can be used as a navigation panel, enabling live playback and exposing the corresponding cell or colony while the cursor is dragged across a graphics plot. An additional event that was thus noted was the identification of a ‘jump-like’ increase in area during outgrowth, which was usually shown to coincide with either the burst of the cell out of the spore coat (Figure 2) or loss of the coat. The analysis is performed in three steps that can directly be invoked from the menu. In the first step, the program automatically detects all phase-bright spores in frame 1 (t = 0) and marks them with non-destructive colored markers. The researcher is free to change the threshold used to detect phase-bright spores and/or to exclude any misjudged objects, manually. In the second step, the marked spores are followed in time to detect any bright-to-dark transition indicating germination, placing a second marker into the corresponding time frame. Germinating spores were marked whenever the intensity of their center region fell below the initially set threshold. The researcher is free to set a suitable threshold based on the microscopical observations. All resulting intensity vs. time plots are stacked into a single file, plus one extra frame holding the superimposition of all plots for quick overview. The difference between settled intensities before and after transition is defined as the ‘drop’ range, and the time needed to drop from 90% to 10% of the entire drop range is used to characterize the transition speed. We define this event as the germination event, generally presumed to cover the uptake of water and expulsion of dipicolinic acid [6]. In the third step, the macro finds the contour of the (out)growing cell/colony in each frame and measures the area in time. All resulting log_2_(area) vs. time plots are stacked into a single file, plus one extra frame holding the superimposition of all plots for quick overview, similarly as for the intensity vs. time plots. The outgrowth time is determined by marking manually the time of first division minus the time of the end of germination. The growth rate function of SporeTracker is used to calculate generation times of each (emerging) cell growing into a microcolony from curve fits within time windows that are defined manually. Generation times were calculated using the time window from time of first division until the end of the linear part of the log_2_(area) vs. time plot. At the time where a cell/colony would touch either another cell/colony or the image boundary, the measurement is stopped. SporeTracker runs in combination with plugin ObjectJ that maintains an integrated spreadsheet-like table that holds linked parameters such as time point and speed of germination as well as growth parameters. Columns in the table can be used for sorting, qualifying and statistical analysis, while rows are actively linked to the cell markers and allow back-and-forth navigation. Manual inspection using the navigation panel is needed to confirm the results.

All separate stages of development from dormant spores to dividing vegetative cells of control spores were compared with those of stressed spores and fitted according to the most appropriate distributions, which were either normal or log normal (data not shown). Differences in variance were tested with *F*-tests. Depending on the results of the *F*-tests the appropriate *t*-tests were performed to test differences in the average. The images from experiments with vegetative cells as a starting point were analyzed to calculate their generation times by using the growth function of SporeTracker as explained above. First, single cells were each marked manually with a single object marker and their contour (area) was measured in time. Next, the generation times of single cells growing into microcolonies were calculated by fitting the linear part of the generated log_2_(area) vs. time plots.

## Results

### Design and Validation of a Novel Closed air-containing Chamber for Live Cell Imaging of Exponentially Growing Cells under Aerobic Conditions

To ensure unbiased growth conditions in which oxygen limitation does not impede optimal growth, we have designed a novel closed air-containing chamber in where the cells/spores are sandwiched between an agarose-medium pad and glass coverslip. This cast has to fulfill four essential criteria: (i) oxygen availability should be sufficient and the distance between the oxygen compartment and the cells should be as short as possible, (ii) sufficient cell culture medium should be available, (iii) water from the culture medium should not evaporate to ensure stable osmolality, and (iv) the cells have to grow in a monolayer, be immobilized and close to the cover glass. Figure 1 shows the schematic picture of the top and side view of the chamber that satisfies all above-mentioned criteria. Cells/spores are sandwiched between the glass coverslip and a thin (160 µm) agarose-medium pad to ensure immobilization and sufficient culture medium (criteria i and ii). A spacer (Gene Frame®) between the glass coverslip and the glass slide forms a small closed compartment filled with air preventing evaporation of water from the agarose-medium pad (criteria iii and iv). The here-described chamber allowed long term time-lapse imaging with phase-contrast microscopy.

**Figure 3:**
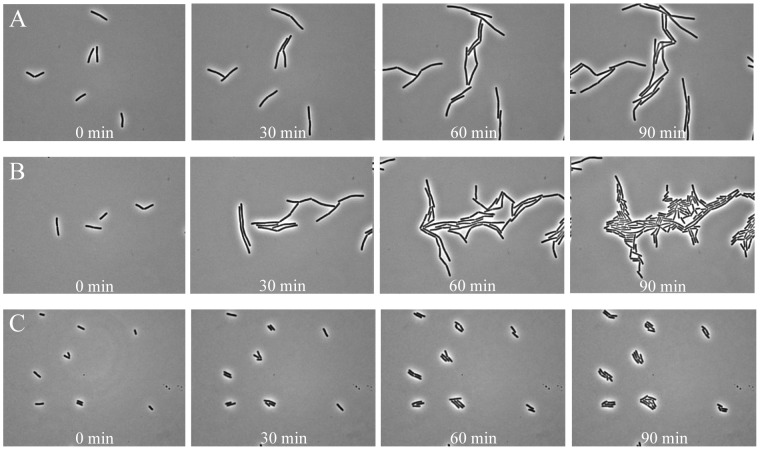
Time-resolved images showing growth and division of *B. subtilis* 1A700 vegetative cells using different media. Exponentially growing cells were spotted on 2.5% complex medium (TSB and LB shown in panel **A** and **B**, respectively) and 100% defined minimal (MOPS-buffered) medium (**C**), and followed in time using phase-contrast microscopy.

To validate the efficiency of the closed air-containing chamber, the generation time of an aerobic bacterium (*B. subtilis* 1A700 vegetative cells) monitored by phase-contrast microscopy was compared with the generation time of the same cells grown in well-aerated shake flasks (Table 1). The time series of vegetative cells were made using 2 type of different culture media that are frequently used in microbiological research: (i) complex, undefined culture medium (TSB and LB), which are often used in microbiological research, including spore germination and outgrowth experiments [11], [19], and (ii) defined minimal medium (MOPS-buffered) frequently used in molecular physiological characterization experiments [17], [20], [21], [22]. Figure 3A, 3B, and 3C show the phase-contrast still images of the first 90 min from the time series of 481 images (4 h) of growing *B. subtilis* vegetative cells in TSB, LB, and MOPS medium, respectively (see Movie S1, S2, and S3). To be able to observe clear cell division in complex medium, the medium was diluted to 2.5% of its original standard concentration (Fig. 3A and 3B). At higher concentrations of complex medium the cells grow as filaments and no clear division was observed (data not shown). Compared to 2.5% complex medium, cells grown on 100% minimal MOPS medium were much shorter (Fig. 3C). In order to facilitate the image analysis process for the calculation of generation times a program (macro) was written. This program defines an image analysis process in ObjectJ, a plugin for ImageJ. This analysis routine is part of our purposely-developed program “SporeTracker” and measures the area of the growing cells in time (see Materials and Methods). The generation time measurements of single cells grown into microcolonies under the microscope and of cells grown in shake flasks were compared (Table 1). The results clearly show that the average generation times of single vegetative cells growing into microcolonies in rich undefined media are in good agreement with the generation times obtained in well-aerated shake flasks. The average generation times of cells grown under the microscope on complex TSB and LB media were 23.8±2.0 min and 23.6±3.2 min, respectively. These are comparable to the generation times obtained for *B. subtilis* cells grown in shake flasks (22.2±1.1 min for TSB and 21.6±0.9 min for LB). Noticeably, for cells cultured under the microscope on 100% MOPS medium a longer average generation time was measured (68.9±4.3 min) as compared to the generation time measured in shake flasks (54.4±0.6 min). Overall, our results indicate that the novel closed air-containing chamber allows undisturbed growth of the aerobic bacterium during time-lapse observation and the purposely-built automated program allows accurate measurements of generation times of single aerobically growing *B. subtilis* cells.

**Table 1:** Mean generation time of *B. subtilis* 1A700 vegetative cells grown under the microscope and in shake flasks in different media.

Medium	Concentration	Mean generation time (min)^a^	
		Microscope^b^	Shake flask (n = 3)^c^
TSB	2.5%	23.8±2.0 (n = 97)	22.2±1.1
LB	2.5%	23.6±3.2 (n = 91)	21.6±0.9
MOPS	100%	68.9±4.3 (n = 115)	54.4±0.6

a Mean generation time is given including the standard deviation.

b Generation times from individual starting vegetative cells growing into microcolonies under the microscope were calculated using SporeTracker (see Materials & Methods for details). The amount of individual starting cells analyzed and gathered from three independent biological replicates is given in brackets.

c Amount of independent biological replicates is given in brackets.

### Live Cell Imaging and Quantitative Analysis of Heterogeneous Spore Germination and Outgrowth

**Figure 4:**
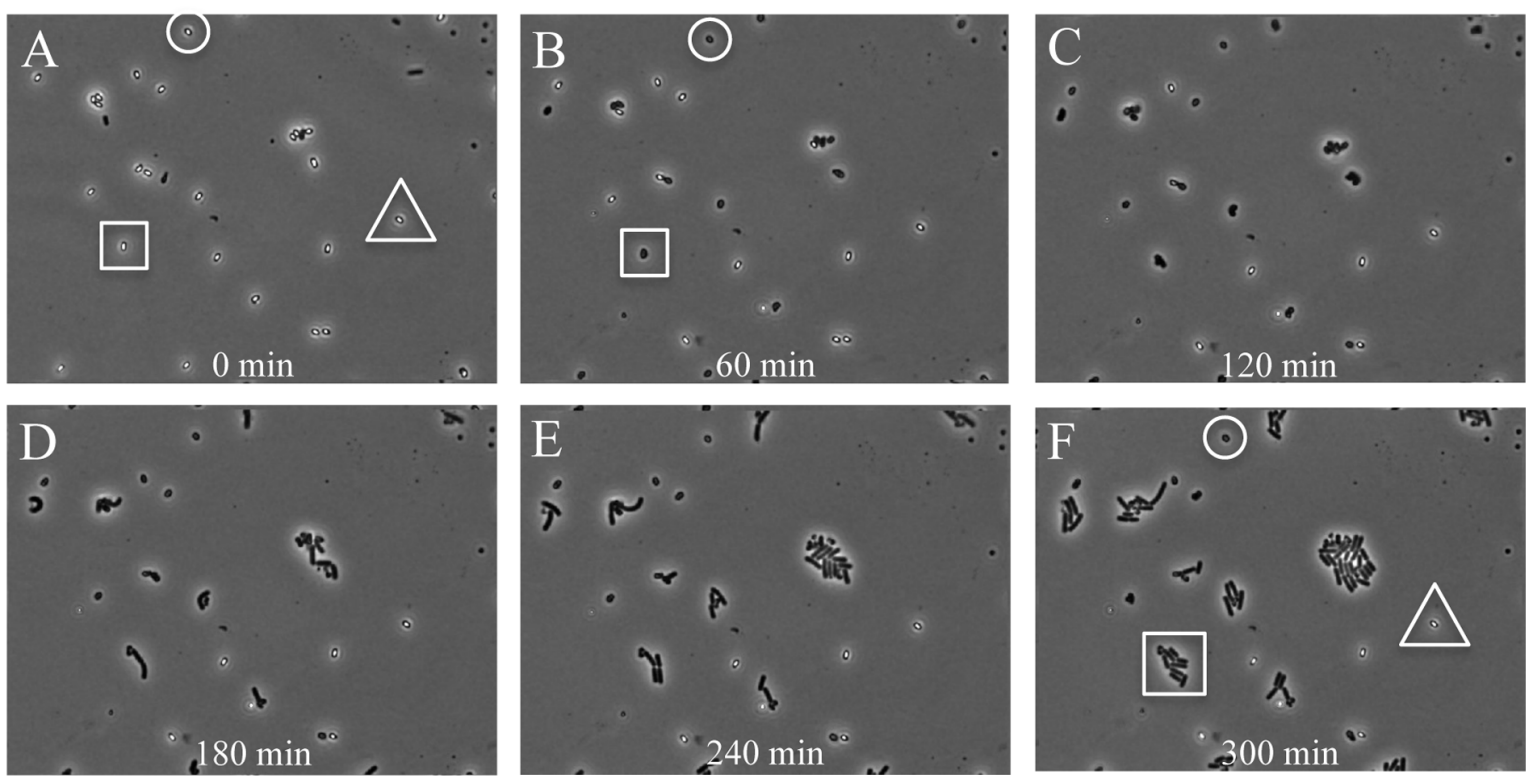
Time-resolved images showing heterogeneous germination and outgrowth of *B. subtilis* 1A700 spores on minimal medium. Heat-activated spores (70°C for 30 min) were spotted on 100% defined minimal (MOPS-buffered) medium including AGFK and followed in time using phase-contrast microscopy. The spore marked in the square (Panel **A**) becomes phase-dark (germinates) within 60 min (Panel **B**), grows out, and forms a microcolony (Panel **F**). The spore marked in the circle (Panel **A**) becomes phase-dark (Panel **B**) but does not grow out within 5 hours (Panel **F**). The spore marked in the triangle (Panel **A**) remains phase-bright throughout the experiment (Panel **F**).

In this study we quantitatively analyze heterogeneity in the time to start of germination, the germination time itself, the outgrowth time, and the time of subsequent cell divisions of *B. subtilis* spores. As concluded above, the closed air-containing chamber is good for the study of dynamics of aerobic bacteria. Although spore germination does not require oxygen, it is a prerequisite for outgrowth and subsequent cell division. In our experiments we spotted 1 µl of a solution of heat-activated (>99.9%) phase-bright spores of *B. subtilis* 1A700 (at a concentration allowing for on average 12 isolated spores per microscopic view) in the above-mentioned chamber and subsequently performed phase-contrast time-lapse microscopy (see Movie S4). Heat-activation of spores (70°C for 30 min) was performed to enhance the initiation of germination, thereby decreasing the degree of heterogeneity, and to kill any remaining cells. Figure 4 shows still images of the first 5 h of germinating and outgrowing spores of *B. subtilis* 1A700 in defined MOPS medium, supplemented with germination triggers 10 mM L-asparagine, 10 mM glucose, 1 mM fructose, and 1 mM potassium chloride (AGFK). The mixture of AGFK is commonly used to induce germination via germination receptors GerB and GerK [6], [23], [24]. Three scenarios were envisaged for subsequent events: (i) spores germinate (become phase-dark) and grow out with time, (ii) spores germinate (become phase-dark), but do not grow out, and (iii) spores remain dormant, meaning that they do not germinate for long time periods even though they are triggered by germinants (super dormant spores). Heterogeneity in spore germination and outgrowth is clearly observed (Fig. 4). The spore marked in the square (Fig. 4A) becomes phase-dark (Fig. 4B), grows out and forms a microcolony within 5 h (Fig. 4F). The spores marked in the circle (Fig. 4A) germinates (Fig. 4B) but does not grow out within the time frame of the experiment (Fig. 4F). The spore marked in the triangle (Fig. 4A) remains phase-bright throughout the 5 h of the experiment (Fig. 4F).

To easily quantify the time of the different stages of germination and outgrowth, and subsequent cell divisions, we developed an in-house semi-automated macro SporeTracker (see Materials and Methods). The germination macro detects the germination step by measuring the decrease in pixel intensity of the spore core and gives a brightness vs. time profile (Fig. 2). Outgrowth and subsequent vegetative growth was measured by measuring in time the increase in the number of pixels with an intensity above background, i.e. measuring the increase in area occupied by outgrowing spores and vegetative cells, respectively (Fig. 2). The obtained results from SporeTracker are discussed below and all individual data can be found in Table S1.

### Effect of a Thermal Stress on Spore Germination and Outgrowth

**Figure 5:**
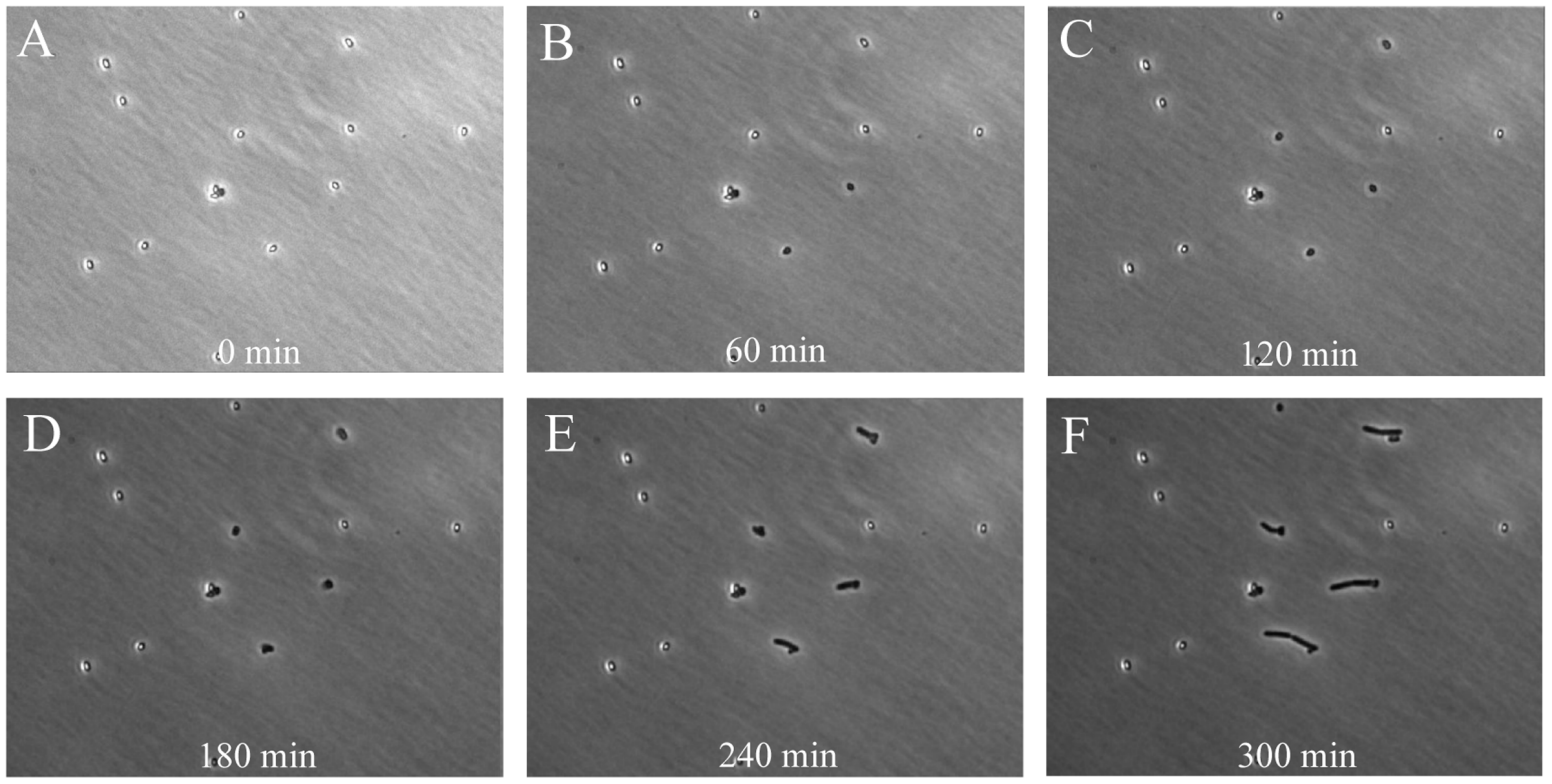
Time-resolved images of germination and outgrowth of heat-treated *B. subtilis* 1A700 spores. Heat-activated spores (70°C for 30 min) were heat-treated for 10 min at 85°C and spotted on 100% defined minimal (MOPS-buffered) medium including AGFK and followed in time using phase-contrast microscopy for 5 hours (Panels **A** till **F**).

**Figure 6:**
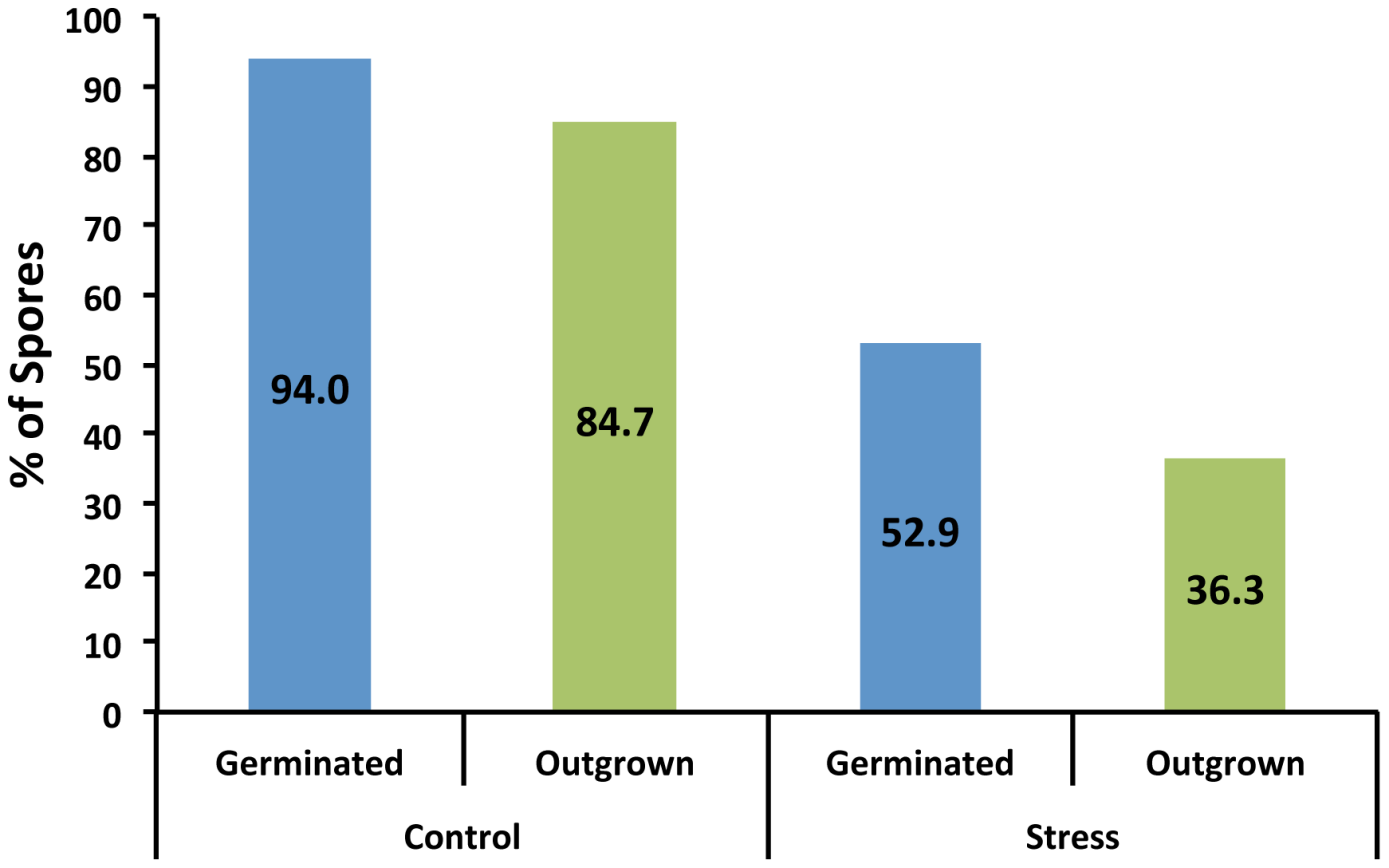
Heat-treated spores show a decrease in the overall number of spores that are able to germinate and grow out within 5 hours. Movies of heat- (85°C for 10 min) and un- treated spores (see Fig. 4 and 5 for details) were analyzed with SporeTracker and the spores were scored (by additional manual inspection) for their ability to germinate and grow out. The total number of spores assessed in the control and stress condition was 218 and 325, respectively.

In order to test our system we assessed the effect of an environmental stress on spore germination and outgrowth. The stress chosen was a thermal pasteurization step, a preservation treatment that is regularly used in the food industry to inactivate vegetative cells. To assess the effect of heat on germination and outgrowth of *B. subtilis* 1A700 spores, heat-activated phase-bright spores were wet-heat-stressed for 10 min at 85°C. The stressed spores were then mounted in the developed chamber and their development was followed over time (see Fig. 5 and Movie S5). Heat-treated spores were still able to germinate and grow out (Fig. 5F), however compared to non-heat-stressed spores, relatively fewer spores seemed to germinate within the time frame of the experiment (Fig. 4F and 5F). Indeed, when all individual data are taken together (Fig. 6), only 52.9% of heat-stressed spores germinated, while 94.0% of non-stressed spores germinated. Additionally, also fewer spores (36.3% vs. 84.7%) grew out when exposed to the thermal stress (Fig. 6).

**Figure 7:**
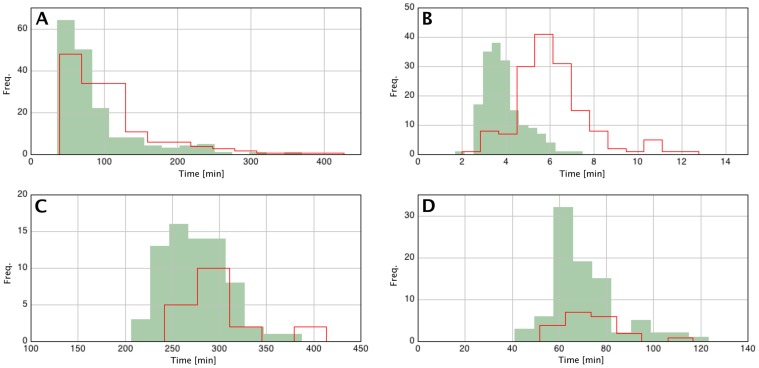
Analysis of individual spores with SporeTracker shows that germination and outgrowth times of heat-treated spores are affected. Movies of heat- (85°C for 10 min) and un- treated spores (see Fig. 4 and 5 for details) were analyzed with SporeTracker. Frequency distributions of heat-treated and control spores were calculated and are shown in red (outline) and green (solid), respectively. Depicted are the frequency distributions of (**A**) the time to the start of germination, (**B**) the germination time, (**C**) the outgrowth time, and (**D**) the generation time of the outgrowing cells into a microcolony.

The different phases of germination, outgrowth, and subsequent vegetative growth were analyzed in detail with SporeTracker. The results show that the heat-treatment caused a significant difference (*P*<0.05) in the average time and the distribution (variance) to the start of germination (Table 2, and Fig. 7A). The average germination speed (time needed to go from a phase-bright to a phase-dark state) was also significantly delayed (*P*<0.05), however the variance of the distribution of the individual germination spores was not different (Table 2, and Fig. 7B). The average initiation of germination in heat-treated spores was delayed by 19 min, and the speed of germination was slowed down by almost 2 min. Although stressed spores seemed to need on average almost 10 min more to grow, this difference was not significant (Table 2, and Fig. 7C). No significant difference was found in the mean doubling time and variance after development to vegetative cells between control and heat-treated spores (Table 2, and Fig. 7D).

**Table 2:** Mean values and standard deviation of different stages of germination and outgrowth of untreated and wet-heat-treated individual *B. subtilis* spores^a^.

Mean ± SD^b^	Treatment	
	None	85°C, 10 min
Start of germination (min)	63±56 (n = 171)	82±68 (n = 152)*
Germination time (min)	3.5±0.9 (n = 171)	5.3±1.7 (n = 151)*†
Outgrowth time (min)	254±34 (n = 72)	263±34 (n = 19)
Generation time (min)	62±14 (n = 87)	62±13 (n = 20)

a Spores of *B. subtilis* 1A700 were wet-heat-treated or not, then heat-activated and germinated in defined minimal (MOPS-buffered) medium including AGFK, and various germination and outgrowth parameters of individual spores were calculated as described in the Materials and Methods.

b Mean time of different stages is given including the standard deviation. The amount of spores/cells analyzed from each stage and gathered from three independent biological replicates is given in brackets. The asterisk indicates that the mean of the distributions between the stress and control experiment are significantly different (*t*-test, *P*<0.05). The dagger indicates that the variance of the distributions between the stress and control experiment are significantly different (*F*-test, *P*<0.05).

Overall, heat-treated spores germinate and grow out less when compared to control spores (Fig. 6). Our analysis further revealed that heat-stressed spores that do germinate are affected in the starting time and duration (both delayed) of the germination process (Table 2, and Fig. 7A and 7B). However, outgrowth and subsequent vegetative cell division does not seem to be significantly affected (Table 2, and Fig. 7C and 7D).

## Discussion

Our newly made closed air-containing chamber described in this paper has proven to be very useful for the observation of growth of single cells of aerobic bacteria and for germination and outgrowth of individual spores. Figure 1 shows the closed chamber, which is made by using a spacer (Gene Frame®). The chamber meets four important criteria for good growth of bacteria and outgrowth of spores. One important criterion is to minimize the evaporation of water from the medium pad at 37°C, thereby maintaining the osmotic balance in the chamber. While the experiments discussed lasted on average around 5 h we have incubated in a preliminary test, the chamber with cells for up to 24 h and observed no drying of the agarose pad with cells (data not shown). Another important criterion of the closed chamber is to ensure sufficient oxygen (air) needed for the growth and outgrowth of bacteria and spores. The oxygen diffuses from the space in the chamber into the pad towards bacterial cells and spores, while these are being observed through the microscope. The third criterion is that the cells or spores are sandwiched between the agarose-medium pad and glass cover slip. This immobilizes the spores/cells on the pad so that they remain in focus throughout the experiment while recording the images and can germinate and grow(out), in monolayer form.

Experiments with vegetative cells showed that identical growth rates could be obtained under the microscope as in well-aerated shake flasks when grown in complex media (TSB, LB) (Table 1). Only in minimal defined MOPS medium a slightly longer generation time was calculated. The reason for this observed difference is until now unclear. The transition from liquid cultures to agar plates of *Escherichia coli* cells grown in rich medium has been described to cause a stress [25]. Perhaps the transition for the cells taken from the pre-culture (grown in shake flasks) to solid medium under the microscope is more stressful when the cells are grown on minimal medium instead of on rich media. This is also exemplified by the relative bigger standard deviation for the average generation time for cells grown on MOPS medium (Table 1). Our experiments with vegetative *B. subtilis* cells showed that the cells have larger sizes when grown in complex medium compared to the defined minimal (MOPS-buffered) medium (Fig. 3). Noticeable too was the observation that higher amount of nutrients present in the complex media lead to fast increase in cell biomass but not a similar increase in cell division (data not shown). As a result, long chains of undivided *B. subtilis* cells (filaments) were formed. This is a common phenomenon when cells are grown in complex solid medium. Therefore, a lower percentage (2.5%) of the original complex medium concentration was used to obtain clear division of cells under the microscope. Weart *et al.* [26] showed that the nutrients dissolved in the culture medium have a strong influence on cell size through their influence on glycolipid biosynthesis. Information on the nutrient concentration is sensed most in that process and stimulates cells to grow in size until the appropriate mass is reached. Weart and Levin suggested that high growth rate delays the tubulin-like FtsZ assembly, the FtsZ ring formation, and subsequent cytokinesis [26], [27], thereby delaying the division. High nutrient levels hence result in a delayed cell division.

*B. subtilis* spores are ubiquitously present in foods, and since they may survive preservation treatments and grow out in end-products, efforts are being made to eliminate or inactivate spores of these bacteria from foods. Moist heat (85°C for 10 min) is routinely used in industry for inactivation in food products of vegetative cells and often leads to spore injury [11]. Hence, before and/or during outgrowth spores must first undergo repair of damage. Both protein denaturation and enzyme inactivation have been associated with spore inactivation by wet heat [8], [28], [29]. Our presented results of wet-heat-treated spores are in line with the observations of Li and co-workers [8] for germination of different *Bacillus* species, and of Stringer *et al.* [13], [14] for germination of *C. botulinum* (Table 2, and Fig. 7). Time to start of germination and germination time were both affected (delayed). Outgrowth and subsequent vegetative growth from cells emerging from heat-treated spores was not significantly changed in terms of the average and frequency distribution. Inflicted damage caused by the heat treatment is likely repaired during and/or before outgrowth, in where the spore becomes metabolically active. Although Stringer *et al.* [13], [14] observed in *C. botulinum* that outgrowth is affected, the heat treatment mainly extended germination and they conclude that damage was quickly repaired and not evident by the time the outgrowing cells started to double.

Noteworthy, when compared to the other investigated processes, the largest variation in the average (standard deviation) is observed in the time to start of germination (Table 2). This might indicate that heterogeneity within the population is intrinsically most apparent in the time to start of germination, *e.g.* due to differences in the amount of germination receptors per spore, rather than in the subsequent stages of germination, outgrowth, and vegetative growth. This reasoning is in line with observations, and their interpretation, reported by Zhang *et al.* [30].

The newly developed program SporeTracker, with its incorporated macros for analyses, allowed for a comprehensive and efficient data analysis. The relevance of our analysis for microbiological risk assessment of foods is significant as it is likely that spores that have turned phase-dark are more likely to become metabolically active and successfully repair damage. Modeling risk of food borne bacterial spores in the context of food safety as well as spoilage is highly topical [31].

To calculate the growth rates of emerging cells from spores and vegetative cells growing into monolayer-microcolonies SporeTracker determines the increase in area over time. Total cell mass of the two-dimensionally growing microcolonies was assumed to be proportional to area including inner gaps, which is only correct for large colonies. However, we felt we could apply this method already after the first cell division, as the exponential curve fit remained typically better than R = 0.99. A disadvantage of determining the growth rate by this method is that not the growth rate of each individual cell within the microcolony is determined, but the average growth rate of the cells from the microcolony as a whole, either started as a single vegetative cell or as a cell emerging from a single spore. Importantly, we calculated in a few randomly picked experiments also growth rates using manually determined cell-lengths of individual cells. These rates were highly similar to the ones obtained by applying SporeTracker (data not shown).

In conclusion, with the use of single-cell analysis techniques we can enhance the mechanistic basis of food preservation and spoilage models targeting bacterial spores. The above-mentioned closed air-containing chamber and image analysis method can be used for assessing the effect of different stresses, *e.g.* different acids and temperature stresses on different stages of germination and outgrowth of the bacteria. Currently we are extending our analyses to include the ratiometric assessment of the dynamics of the internal pH of spores and resulting vegetative cells using pHluorin [32]. In addition, we aim at developing a micro-fluidics system that should allow for the change of growth media while observing the spore population. Such developments should provide the means to study the effect and dynamics of the response of *B. subtilis* spores and cells at the single cell level, upon exposure to *e.g.* the common weak acid preservative sorbic acid.

## Supporting Information

Table S1 **Results obtained from SporeTracker of individual germinating and outgrowing** ***B. subtilis*** **1A700 spores.** Either (A) heat-activated (control) or (B) heat-activated and heat-treated (stress).
(XLS)

Movie S1 **Growth and division of** ***B. subtilis*** **1A700 vegetative cells grown on 2.5% complex TSB medium.**
(AVI)

Movie S2 **Growth and division of** ***B. subtilis*** **1A700 vegetative cells grown on 2.5% cpmplex LB medium.**
(AVI)

Movie S3 **Growth and division of** ***B. subtilis*** **1A700 vegetative cells grown on 100% defined minimal (MOPS-buffered) medium.**
(AVI)

Movie S4 **Germination and outgrowth of heat-activated** ***B. subtilis*** **1A700 spores in 100% defined minimal (MOPS-buffered) medium including AGFK.**
(AVI)

Movie S5 **Germination and outgrowth of heat-activated and heat-treated (10 min at 85°C)** ***B. subtilis*** **1A700 spores in 100% defined minimal (MOPS-buffered) medium including AGFK.**
(AVI)
